# Parameters Influencing Baseline HIV-1 Genotypic Tropism Testing Related to Clinical Outcome in Patients on Maraviroc

**DOI:** 10.1371/journal.pone.0125502

**Published:** 2015-05-13

**Authors:** Saleta Sierra, J. Nikolai Dybowski, Alejandro Pironti, Dominik Heider, Lisa Güney, Alex Thielen, Stefan Reuter, Stefan Esser, Gerd Fätkenheuer, Thomas Lengauer, Daniel Hoffmann, Herbert Pfister, Björn Jensen, Rolf Kaiser

**Affiliations:** 1 Institute of Virology, University of Cologne, Cologne, Germany; 2 Department for Bioinformatics, University of Duisburg-Essen, Essen, Germany; 3 Computational Biology and Applied Algorithmics, Max Planck Institute for Informatics, Saarbrücken, Germany; 4 Department of Gastroenterology, Hepatology and Infectiology, University Hospital of Düsseldorf, Düsseldorf, Germany; 5 Department of Dermatology, University of Duisburg-Essen, Essen, Germany; 6 First Department of Internal Medicine, University of Cologne, Cologne, Germany; University of Rome Tor Vergata, ITALY

## Abstract

**Objectives:**

We analysed the impact of different parameters on genotypic tropism testing related to clinical outcome prediction in 108 patients on maraviroc (MVC) treatment.

**Methods:**

87 RNA and 60 DNA samples were used. The viral tropism was predicted using the geno2pheno_[coreceptor]_ and T-CUP tools with FPR cut-offs ranging from 1%-20%. Additionally, 27 RNA and 28 DNA samples were analysed in triplicate, 43 samples with the ESTA assay and 45 with next-generation sequencing. The influence of the genotypic susceptibility score (GSS) and 16 MVC-resistance mutations on clinical outcome was also studied.

**Results:**

Concordance between single-amplification testing compared to ESTA and to NGS was in the order of 80%. Concordance with NGS was higher at lower FPR cut-offs. Detection of baseline R5 viruses in RNA and DNA samples by all methods significantly correlated with treatment success, even with FPR cut-offs of 3.75%-7.5%. Triple amplification did not improve the prediction value but reduced the number of patients eligible for MVC. No influence of the GSS or MVC-resistance mutations but adherence to treatment, on the clinical outcome was detected.

**Conclusions:**

Proviral DNA is valid to select candidates for MVC treatment. FPR cut-offs of 5%-7.5% and single amplification from RNA or DNA would assure a safe administration of MVC without excluding many patients who could benefit from this drug. In addition, the new prediction system T-CUP produced reliable results.

## Introduction

To enter the host cell, the Human Immunodeficiency Virus type 1 (HIV-1) binds to the cellular receptor CD4 and one of the cellular coreceptors CCR5 or CXCR4. Since MVC binds exclusively to the CCR5 molecule, its administration must be preceded by a coreceptor usage (or tropism) phenotypic or genotypic analysis. Among the phenotypic assays, the most-widely used is the Enhanced Trofile (ESTA) test, whose validity has been shown in the MOTIVATE, MERIT and A4001029 trials [1–3], although other methods are also currently available [4]. In the genotypic approaches, the viral tropism is predicted from the viral third hypervariable loop of the viral gp120 (V3) sequence. The most widely-used sequencing method is the bulk (also referred to as Sanger or population-based) sequencing, which is now the standard of care in determining initial antiretroviral treatments for new patients and optimising changes upon therapy failure. In addition, several studies have demonstrated that the V3 bulk sequencing produces tropism results comparable to ESTA and is adequate for clinical purposes [5–7]. A new genotypic approach is the so-called next generation sequencing (NGS), a term applied to a variety of sequencing platforms which allow a deeper resolution in the quasispecies detecting minority viral subpopulations with prevalences down to 1%. To date, NGS data have primarily been applied in research context although first reports have shown its utility for clinical purposes [8–13]. Sequences generated in genotypic testing (bulk and NGS) need to be interpreted by bioinformatics tools to produce a tropism prediction. For population-based sequencing, geno2pheno_[coreceptor]_ is the recommended tool for genotypic tropism testing in both the Austrian-German and the European tropism testing guidelines [14,15]. For NGS, the geno2pheno_[454]_ tool is freely-available within the geno2pheno system on the internet.

Different diagnostic parameters affecting the tropism prediction reliability are currently under debate. Limited data have been published regarding the impact of FPR cut-offs<20%, use of viral RNA versus proviral DNA samples, single versus triple amplification, and clinical relevance of MVC-resistance mutations on population-based tropism prediction related to clinical outcome under MVC treatment. In this study, we have evaluated the performance of two independent systems: geno2pheno_[coreceptor]_, whose predictions are mainly based on geometric distances of amino acid pairs within the structure of V3 [16], and T-CUP, which performs its predictions by analysing conformational and hydrophobicity properties of the V3 loop [17], on 108 patients treated with MVC. Our results will help in elucidating the best testing settings in order to achieve a successful treatment response in a maximum number of patients.

## Methods

All patients attending Düsseldorf, Cologne and Essen-Duisburg University Hospitals and treated with MVC were included in this non-interventional and retrospective study. Both plasma RNA and proviral DNA (when available) were analysed with bulk sequencing [18].

The viral tropism was predicted with the geno2pheno_[coreceptor]_ (clonal setting) and T-CUP tools, using different FPR cut-offs: 1%, 3.75%, 5%, 7.5%, 10%, 15% and 20%. All samples with enough remaining material were additionally analysed with the ESTA assay, NSG [19], and/or in triplicate. In the NSG analysis, samples were analysed with the geno2pheno_[454]_ tool and classified as X4 when more than 2% of the sequences displayed a FPR value above the considered cut-off.

CD4 counts, VL, and therapies were collected. Therapy success was defined as a decrease in VL≥2 logs with respect of baseline or VL<50 copies/mL at two consecutive sampling times. Positive Predictive Value (PPV) was calculated as the percentage of patients carrying baseline R5 viruses who succeeded under MVC treatment. The number of active drugs in the concomitant optimised background therapy (genotypic susceptibility score; GSS) was calculated with the prediction tools geno2pheno [20] (http://www.genafor.org; for protease-, reverse transcriptase-inhibitors, and raltegravir) and HIV-GRADE (http://www.hiv-grade.de; for enfuvirtide) for all the patients where PR/RT and IN sequences were available. The output of the algorithms was mapped to numerical values: susceptible = 1 (or 0.5 for NRTIs), intermediate = 0.5 (0.25 for NRTIs) and resistant = 0.

Correlation analysis between clinical parameters and therapy response were performed using Fisher’s exact test. Differences of VL and CD4 between responders and non-responders during the MVC therapy were assessed using the t-test. All reported values are two-sided. We used a logistic regression model to analyze multivariate dependencies. The response to MVC was used as the dependent variable and tropism predictions by geno2pheno_[coreceptor]_ and T-CUP, as well as viral load, CD4 cell counts, nadir CD4, GSS, number of previous therapies and number of previous virological failures as independent variables. Prediction performance of the logistic regression model was evaluated by leave-one-out cross-validation. The level of significance was a p-value ≤0.05.

All data in this study were analyzed anonymously. All data used in this study were approved by the Ethics Committee of the University Hospital Duesseldorf (No. 1733).

## Results

### Baseline clinical characteristics

**Fig 1:**
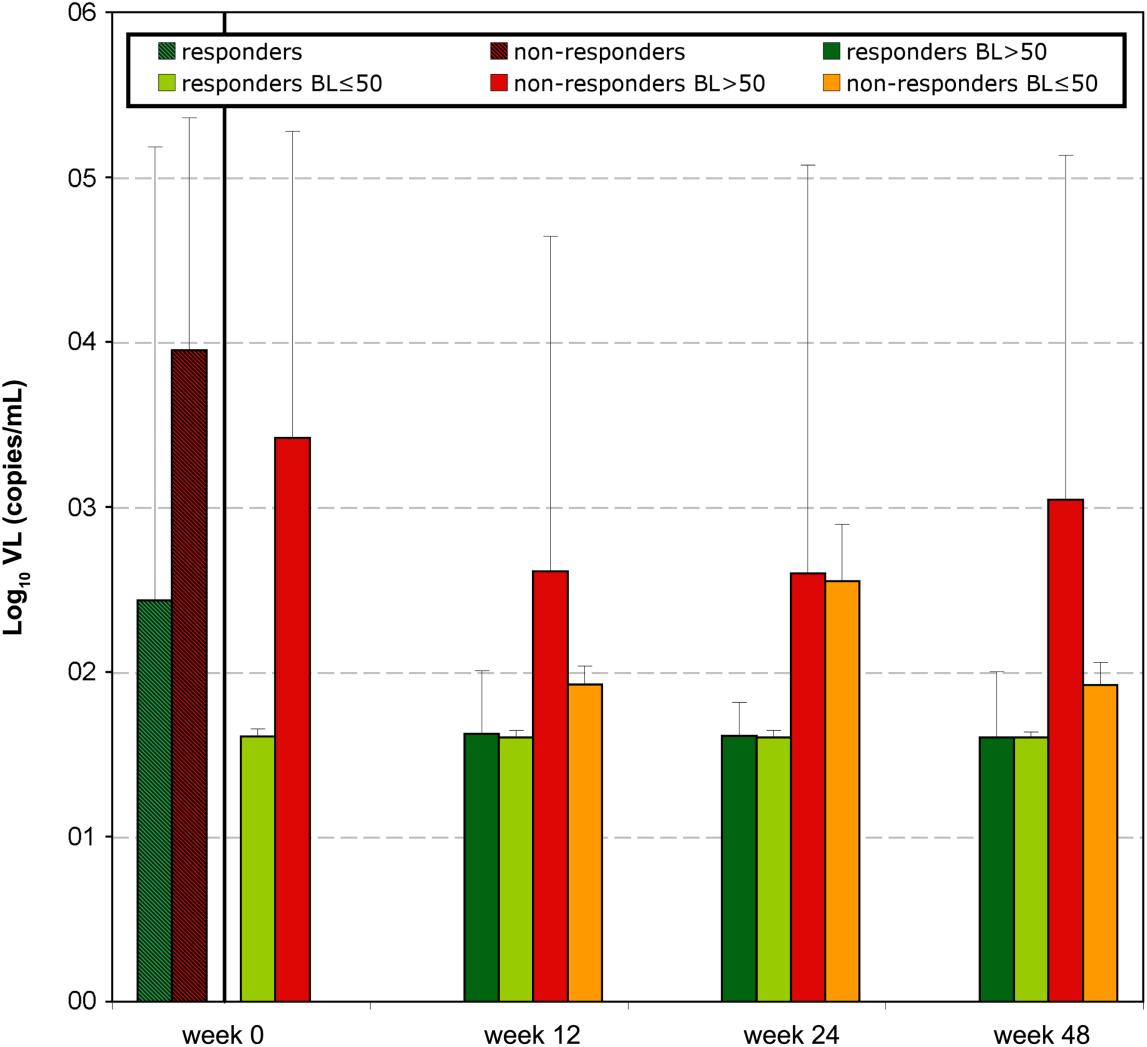
Viral load follow-up of the 108 patients. Depicted data correspond to VL mean values with standard deviations. Week 0: data from 97 patients who subsequently responded to treatment (responders) are represented in striped green; data from 11 patients that subsequently did not respond to treatment at any time (non-responders) are represented in striped red. Week 0, 12, 24, 48: responding patients: data from patients responding to the therapy at the corresponding sampling date are displayed in green: those who started MVC therapy with undetectable VL in light green, and those with baseline (BL) VL above detection limit in dark green; non-responding patients: data from patients not responding to the therapy at the corresponding sampling date are displayed in red (those with detectable baseline VL) and in orange (those with undetectable baseline VL).

**Fig 2:**
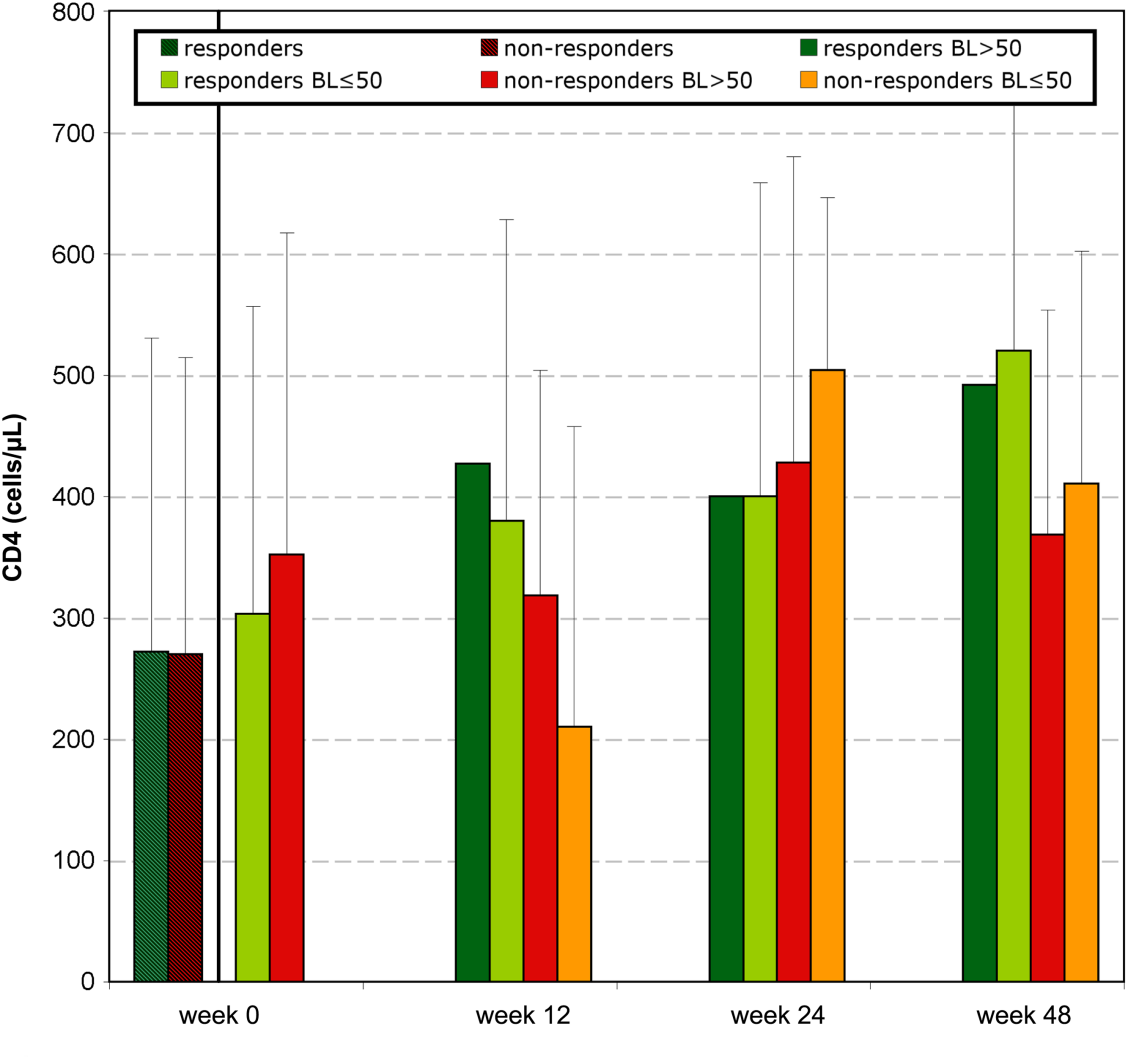
CD4 follow-up of the 108 patients. Depicted data correspond to CD4 mean values with standard deviations. Week 0: data from 97 patients who subsequently responded to treatment (responders) are represented in striped green; data from 11 patients that subsequently did not respond to treatment at any time (non-responders) are represented in striped red. Week 0, 12, 24, 48: responding patients: data from patients responding to the therapy at the corresponding sampling date are displayed in green: those who started MVC therapy with undetectable VL in light green, and those with baseline (BL) VL above detection limit in dark green; non-responding patients: data from patients not responding to the therapy at the corresponding sampling date are displayed in red (those with detectable baseline VL) and in orange (those with undetectable baseline VL).

**Fig 3:**
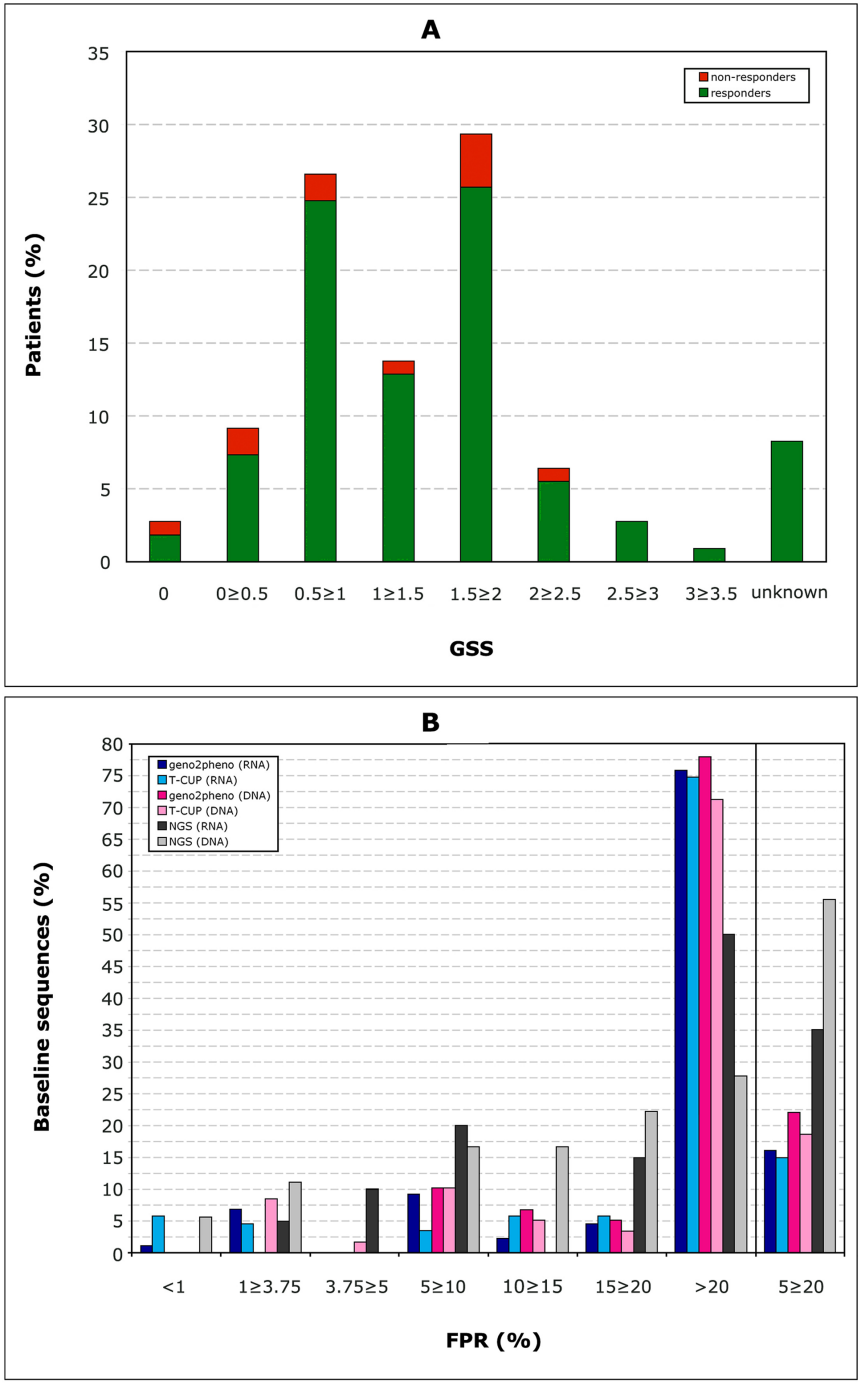
Baseline characteristics. Panel A: Number of active drugs in the GSS. Panel B: Distribution of the FPR of the baseline viruses: 87 RNA and 60 DNA sequences with single population-based sequencing, and 20 RNA and 18 DNA samples analysed by NGS.

162 patients were initially enrolled in this study, whereof 108 were finally included in the analysis as at least one baseline V3 sequence and clinical follow-up on MVC treatment were available. 88/108 were male. The median age was 44 years (range 18–81), patients were in median 17 (2–24) years infected with HIV, 9 patients were therapy-naive and 99 patients had undergone a median of 8 (1–25) previous antiretroviral therapies; the 99 therapy-experienced patients had experienced a median of 2.5 (0–25) virological failures. The median VL was 320 copies/mL (40–1265000), whereof 31 patients started the MVC-therapy with a VL≤50, and 77 with detectable viremia [median 829 copies/mL (52–1265000)] (Fig 1). The median CD4 counts was 398 cells/μL (10–1430), [median of 370 (100–1240) for patients starting MVC with VL≤50; median of 370 (10–1250) for patients starting MVC with detectable VL] (Fig 2). The median nadir CD4 counts was 105 cells/μL (1–446) [median of 83 (1–334) for patients starting MVC with VL≤50; median of 111 (7–446) for patients starting MVC with detectable VL]. Clinical follow-up was available for a median of 53 weeks (12–131). The baseline GSS could be calculated for 100/108 (92.6%) of the patients (Fig 3, panel A). It included in median 1.5 active drugs (range 0–3.5).

87 RNA and 60 DNA baseline sequences were obtained and their FPR calculated (Fig 3, panel B). 43 samples were analysed with the ESTA assay, whereof 31 were classified as R5, 6 as X4 (D/M), and 6 produced a negative report. 34 RNA and 21 DNA samples were analysed with NGS. The median VL for the input was 5043 copies/mL (40–750000), and the number of reads 1158 (381–2050). All baseline tropism data were submitted to the treating physician who finally took the decision whether to start a MVC-containing therapy.

97/108 (89.8%) patients achieved therapy success under MVC treatment (responders). The earliest sampling point for success was in median 13 weeks under MVC (range 4–28). Baseline VL was lower for the 97 responders (median = 280; range 40–1265000) than for the eleven non-responders (8914; 50–750000), although no correlation between baseline VL and response to treatment was found (Fig 1). At week 12, 24 and 48 VL values from responders were significantly different to the corresponding non-responders (p≤0.009). On the other hand, baseline CD4 was higher for the 97 responders (median = 321; range 18–1430) than for the eleven non-responders (270; 10–820), and baseline CD4<300 cells/μl correlated with therapy failure (p = 0.039) (Fig 2). CD4 counts were not significantly different between responders and non-responders at week 12, 24 or 48 and were, in median, above the baseline level.

The univariate analysis of baseline parameters showed that baseline VL≤66 copies/mL (p = 0.005), baseline CD4>120 cells/μL (p = 0.008), and nadir>25 cells/μL were predictors of therapy success; GSS, and the number of previous therapeutic regimens and virological failures were not predictors of treatment outcome. The multivariate analysis of baseline parameters showed no significant associations, except for viral load and nadir CD4 count (p = 0.0458 and p = 0.0405, respectively). However, these associations were rather weak (-9.204e-07 and 7.786e-04). These results are also reflected in the prediction performance in a leave-one-out cross-validation, where the logistic regression model was not able to accurately predict MVC response (AUC = 0.611). Thus, the results from the multivariate analysis are in agreement with those from the univariate analyses.

### Tropism prediction concordance among the three methods

**Fig 4:**
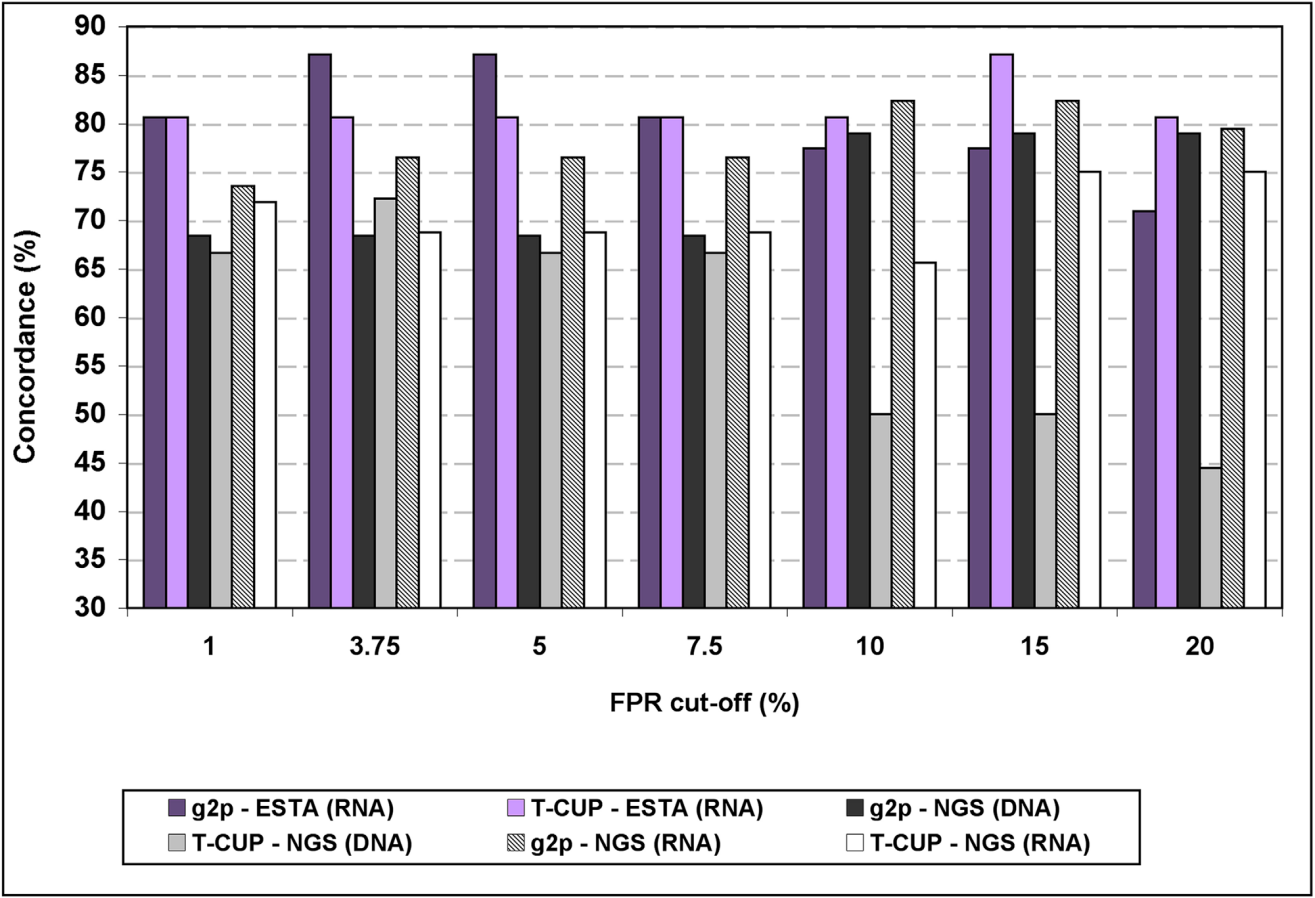
Concordance between the genotypic, ESTA and NGS results. Values were calculated using 37 samples for ESTA comparison and 45 samples for NGS analysis. g2p: geno2pheno_[coreceptor]_.

We analysed the concordance in tropism prediction between population-based sequencing, ESTA and NGS (Fig 4). The concordance between ESTA and bulk sequencing with subsequent geno2pheno_[coreceptor]_ interpretation was in average 80.2% (range 71.0%-87.1%), and between ESTA and T-CUP analysis 81.6% (80.6%-87.1%). Concordance was very similar for all the FPR cut-offs tested.

For NGS, concordance with bulk sequencing with geno2pheno_[coreceptor]_ was in average 76.7% (71.7%-81.1%), and with T-CUP 66.3% (60.0%-70.0%). Predictions by NGS and bulk sequencing from RNA samples accorded more [average 78.2 (geno2pheno) and 70.5 (T-CUP)] than those from DNA samples [median 72.9 (geno2pheno) and 59.5 (T-CUP)] did. Concordance with NGS was also higher at lower FPR cut-offs.

### Baseline tropism related to clinical outcome

#### Influence of baseline FPR cut-off on PPV and number of patients eligible for MVC

Baseline R5 tropism prediction by geno2pheno_[coreceptor]_ from both RNA and DNA samples associated with therapy success. For the 87 RNA samples, statistical correlations between baseline R5 tropism and successful treatment were detected with the geno2pheno-FPR cut-off = 5%(p≤0.039), and T-CUP-FPRs>5% (p≤0.041). For the 60 DNA samples, significant correlations were found with the geno2pheno-FPR cut-offs = 5% and 20% (p≤0.023), and all tested T-CUP-FPRs (p≤0.012). Classification of baseline sample as R5 by ESTA and NGS (<2% of the sequences with FPR≥10%) also correlated with therapy success (p = 0.023 and 0.025, respectively). Baseline R5 detection using the FPR the cut-off = 0% (equivalent to no tropism testing as all samples are classified as R5) for bulk and NGS analyses did not correlate with therapy success.

**Fig 5:**
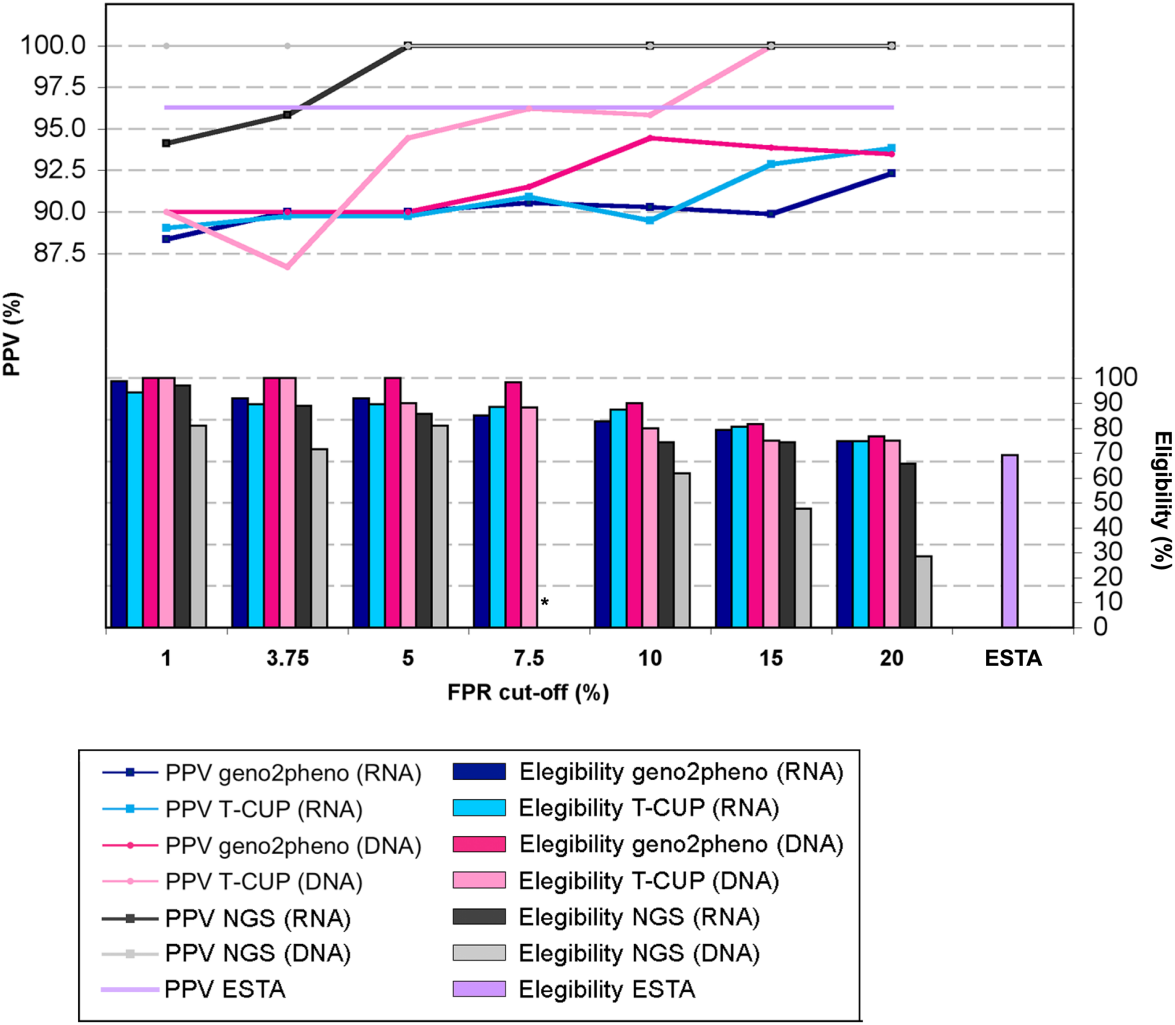
Influence of FPR cut-offs on PPV and number of patients elegible for MVC treatment. Values were calculated using all available RNA (n = 87) and DNA (n = 60) samples; ESTA (n = 37); NGS (n = 45). * No NGS values for the NGS FPR cut-off of 7.5% were available.

The PPV for all patients with predicted baseline R5 viruses was around 90% (Fig 5), without significant differences depending on the FPR cut-off, the sample material (RNA or DNA) and the method used. In addition, concomitant baseline RNA and DNA samples were available for 39 patients. Baseline tropism predictions were concordant in median 87.2% (range 100%-76.9%) for geno2pheno_[coreceptor]_ and 80.5% (69.2%-100%) for T-CUP. Three patients displayed high discordances between the FPR values from the concomitant RNA and DNA. Patients #96 [RNA: 75.9% (geno2pheno_[coreceptor]_), 68.3% (T-CUP); DNA: 6.6% (geno2pheno_[coreceptor]_), 3.2% (T-CUP)] and #105 [RNA: 41.2% (geno2pheno_[coreceptor]_), 17.1% (T-CUP); DNA: 7.9% (geno2pheno_[coreceptor]_), 2.0% (T-CUP)] were non responders. Patient #145 [RNA: 7.0% (geno2pheno_[coreceptor]_), 2.2% (T-CUP); DNA: 47.1% (geno2pheno_[coreceptor]_), 58.7% (T-CUP)] achieved undetectable VL at week 18 and remained so until week 43, the last VL value available to us.

The number of patients carrying baseline R5 viruses decreased as the FPR cut-off increased. The FPR cut-off = 5% classified an average of 6.6% more patients as R5 carriers compared to FPR cut-off = 10%, and 17.3% more patients when compared to FPR cut-off = 20% (Fig 5).

#### Triple amplification of the samples

Triplicate amplifications of 27/31 RNA and 28/33 DNA samples were achieved and their tropism correlated with the clinical outcome. Viruses were considered R5 when all three sequences produced a FPR value above the specific cut-off. For concordance comparison with single amplification (singleton), the first amplification of each sample was used as control.

Detection of baseline R5 tropism associated with therapy success in control and triple amplifications. For the controls, significant correlations between baseline R5 tropism and therapy success were found for RNA samples [geno2pheno-FPR cut-off = 5% (p = 0.049); T-CUP FPR cut-offs>5% (p≤0.049)]. For DNA samples, correlations were found with T-CUP-FPR cut-offs>7.5% (p≤0.022). These results are concordant with those obtained with the whole dataset of 87 RNA and 60 DNA sequences previously described. For the triplicates, significant correlations were found for RNA samples [geno2pheno-FPR cut-off = 5% (p = 0.013); T-CUP-FPR cut-offs = 7.5%-10% (p≤0.046)], and for DNA samples with T-CUP-FPR cut-off = 7.5% (p = 0.035).

**Fig 6:**
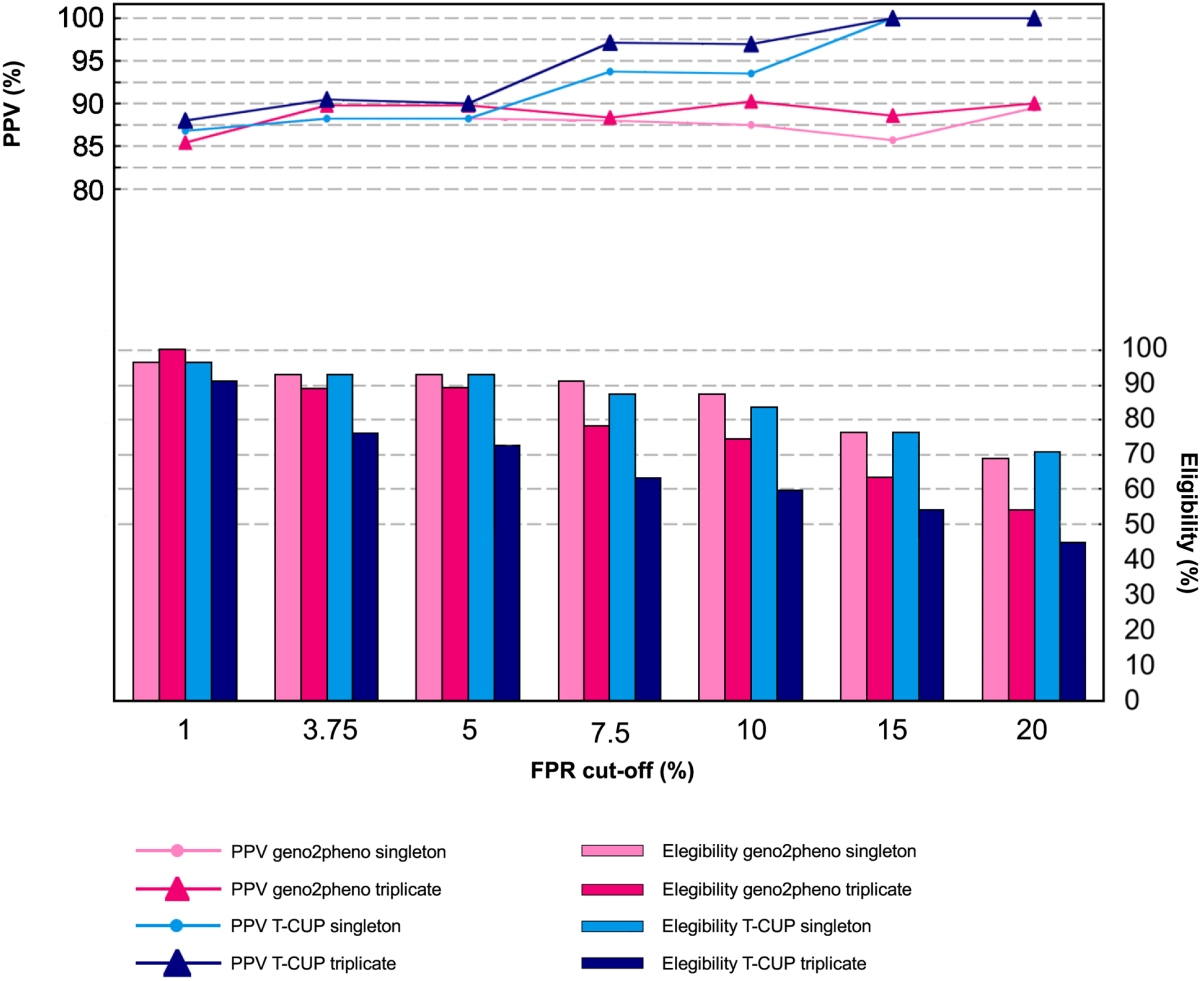
Influence of FPR cut-offs and triple amplification on PPV and number of patients elegible for MVC. Depicted data correspond to the 27 RNA plus 28 DNA samples that could be amplified in triplicate.

The PPVs of triple amplifications were close to 90% and not significantly superior to the corresponding singleton control, independently of the type of sample or FPR cut-off used (Fig 6). The use of triple amplification further reduced the number of baseline R5 viruses in median 18%, but up to 37.5%. Adding together the effects of FRP cut-off setting and triple amplification, a median of median 6.2% (3.6%-46.2%) patients would be excluded from MVC treatment when using FPR cut-off of 10% and triple amplification compared to using FPR cut-off = 5% and single amplification.

### Clinical characteristics and viral tropism at MVC failure

#### Detection of X4 viruses

From 19 patients failing their MVC therapy, 24 samples after virological rebound could be sequenced (Table 1). Marked decreases in FPR were detected in 2/24 (8.3%)- 4/24 (16.7%) (geno2pheno_[coreceptor]_ and T-CUP, respectively) of the sequences from failing patients (patients #31, #83, #85, #108). Increases of FPR were detected in 3/24 (12.5%)- 5/24 (25.0%) sequences (#21, #87, #103, #105, #109). X4 sequences were found in 2/24 (8.3%)- 9/24 (37.5%) (geno2pheno_[coreceptor]_, cut-off = 3.75%—cut-off = 20%, respectively) or 4/24 (16.7%)– 9/24 (37.5%) (T-CUP; cut-offs 3.75%-20%, respectively) of the sequences. Additionally, 19 samples from 16 patients with a successful MVC treatment were available. 2/19 (10.5%) (geno2pheno and T-CUP) of the samples displayed a FPR<3.75%, 3/19 (15.8%)- 1/19 (5.3%) (geno2pheno—T-CUP, respectively) showed FPRs between 5%-20%, and 14/19 (73.7%) -16/19 (84.2%) FPRs≥20%. Based on these data, therapy failure did not correlate with the detection of X4 viruses after virological rebound, at any of the FPR cut-offs tested.

**Table 1:** Viral characteristics after treatment failure/success.

		Baseline					After treatment failure			
Pat. ID	Comments	Sample material	g2p FPR	T-CUP FPR	ESTA	GSS	Sample material	g2p FPR	T-CUP FPR	GSS
1		RNA	38.0	33.1	NA	1.5	RNA	15.0	43.9	2.0
		DNA	21.5	20.2	NA	1.5	DNA	34.4	22.4	2.0
19		RNA	43.0	23.5	NA	0.0	RNA	69.8	38.8	0.0
21		RNA	0.2	0.0	NA	2.0	DNA	12.0	2.1	2.0
31^a^		RNA	40.3	11.3	NA	0.5	DNA	2.6	2.5	2.0
42		DNA	85.6	68.2	NA	2.0	DNA	87.1	62.9	2.0
48		RNA	21.2	82.6	R5	2.0	DNA	27.3	55.8	2.0
58		RNA	67.9	18.2	NA	1.0	DNA	78.1	22.4	n. a.
65		RNA	96.5	39.7	R5	2.0	RNA	94.6	58.6	n. a.
		DNA	88.8	77.2	NA	2.0	-	-	-	-
66	B.A.T.	RNA	85.8	22.8	R5	2.0	RNA	84.8	18.1	2.0
		DNA	42.6	89.9	NA	2.0	-	-	-	-
83	B.A.T.	RNA	91.2	74.1	NA	1.5	DNA	6.0	1.8	1.75
85		DNA	89.1	68.3	R5	1.0	RNA	64.0	5.3	1.0
87	B.A.T.	RNA	64	5.3	NA	2.0	RNA	67.9	50.2	2.0
		DNA	76.9	6.1	NA	2.0	-	-	-	-
103	B.A.T.	RNA	96.1	35.4	NA	2.25	-	-	-	-
		DNA	47.7	9.3	NA	2.25	DNA	71.5	65.0	2.5
105		RNA	50.5	17.1	NA	2.0	-	-	-	-
		DNA	7.9	2.0	NA	2.0	DNA	24.4	11.7	2.0
108^a^		RNA	2.8	10.3	D/M	1.0	RNA	0.2	0.4	0.0
109^a^	S.B.A.T.	RNA	6.8	3.2	D/M	1.75	RNA	50.5	17.1	3.0
		DNA	17.1	3.6	NA	1.75	DNA	16.6	11.4	3.0
123	B.A.T.	RNA	79.5	42.0	NA	2.0	RNA	56.9	32.9	2.0
		DNA	80.0	52.6	NA	2.0	DNA	82.7	90.2	2.0
152		RNA	6.0	33.5	NA	0.5	DNA	5.7	30.2	1.25
							RNA	4.6	35.5	1.25
164	B.A.T.	RNA	16.3	50.0	NA	1.0	RNA	26.8	50.6	1.0
							DNA	16.3	43.6	1.0

^a^non-responders. The other patients failed at week 24 or 48.

g2p: geno2pheno_[coreceptor]_

GSS: genotypic susceptibility score

ESTA: Enhanced Trofile assay

NA: not available

B.A.T: reported-bad adherence to MVC-treatment

S.B.A.T: suspected-bad adherence to MVC-treatment

#### GSS

Changes in the GSS after failure were detected in 7 out of the 17 (41.2%) of the failing patients whose GSS could be calculated (Table 1). In 6/17 (35.3%) patients (#1, #31, #83, #103, #109, #152) the number of active drugs in the OBT increased with the respect to the baseline, while for patient #108 it decreased to zero. No GSS could be calculated for patients #58 and #65 due to unsuccessful amplification of the RT/PR region. 9 PR/RT sequences could be obtained from the 16 succeeding patients. The GGS increased in one patient, remained like at baseline in 5 patients, and decreased in 3 cases compared to those at the baseline.

No correlation between virological rebound and the GSS on MVC therapy or the duration of the treatment was detected.

#### MVC resistance-associated mutations

The mutations 4L, 11SR, 13HS, 18G, 19ST, 20F, 21I, 22T, 25D, 26V, INS 15, INS24, DEL18, and the combinations 11S+26V, 18G+22T, 19ST+26V, 20F+25D+26V, and 20F+21I have been related to MVC resistance independent of CXCR4 tropism [21,22]. We analysed their prevalence in the baseline sequences, the 18 samples after MVC failure, and the 14 samples during successful treatment.

99.3% sequences displayed at least one of these mutations. The prevalence of these sequences at baseline and after MVC failure was very similar: 4L, 13S, 18G, 19S, 21I, 25D, and 26V were detected in ≤10% of the samples; 19T and 22T in 10%-30%; 11S, 13H, 20F and 25D in ≥48% of the sequences. No mutation or mutational pattern at baseline or after MVC failure correlated with viral rebound.

## Discussion

The usefulness of population-based sequencing followed by use of the geno2pheno_[coreceptor]_ tool for tropism prediction in clinical settings has been extensively demonstrated [5–7,23–25]. Genotypic tropism testing may be decentralised, is faster and cheaper, and has become the most wide-spread mode of testing in Europe. Several studies have analysed the concordance of different genotypic tropism prediction tools with ESTA and NGS results. However, for clinical purposes, tropism assays should predict the usefulness of CCR5 antagonists to predict therapy outcome in HIV routine daily practice, and not just produce intra-assay similarity. Hence, data on patients receiving MVC-containing therapies, tested genotypically, and with available clinical follow-up are essential. Our analysis is, so far, the largest attempt to simultaneously explore the influence of FPR cut-off, use of proviral DNA, triple amplification, and analysis of MVC resistance mutations on tropism prediction and clinical outcome on patients from HIV care centres. This analysis included 108 patients from German HIV day units, therefore reflecting the current treatment situation in Germany, and probably in most Western Europe. It further corroborates the reliability of genotypic analysis with geno2pheno_[coreceptor]_, ESTA and NGS for tropism prediction in clinical settings and presents the first genotypic results of the T-CUP tool on clinical samples.

The key aspect for the accuracy of the genotypic tropism prediction is the FPR cut-off, the numerical value up to which a virus is considered R5. A too high cut-off will lead to MVC prescription to very few patients who surely carry R5 viruses but will also exclude many others who probably would benefit from it. On the other hand, too low cut-offs will allow MVC prescription to many more patients, some of them perhaps carrying X4 viruses. The results presented here support the necessity of tropism determination prior to MVC administration, as baseline R5 detection with control FPR cut-off = 0 (equivalent to no-testing) was not a predictor of therapy success, contrary to baseline R5 detection with cut-offs = 5% (for geno2pheno_[coreceptor]_) or >5% (T-CUP), which correlated with therapy success. Besides, our study showed that predictions from RNA samples were concordant to the predictions from concomitant DNA and that the same FPR cut-offs may be applied [26–30]. Additionally, we also demonstrate that baseline FPR cut-off = 20% do not represent an improvement to prevent treatment failure compared to lower values, as that high FPR cut-offs reduced up to 20% the number of patients eligible for MVC treatment but do not significantly increase the PPV. In fact, both T-CUP and geno2pheno_[coreceptor]_ have been developed to identify X4 viruses with highest sensitivity, so the use of too high FPR cut-offs should greatly overestimate X4 variants. Supporting our results, increasing evidence published in the latest years have also suggested the use of FPR cut-offs ranging 5.75%-10% in order to avoid exclusion of patients who would succeeded in their MVC treatment, while still guaranteeing a save administration of the drug [6,7,23,27,30,31].

A major concern for MVC treatment is a possible failure through tropism switch due to outgrowth of X4 variants not detected at baseline testing. The detection limit for population-based sequencing is 10%-20%, depending on the technical expertise and the input viral copy number [32,33]. To reduce this limitation, triple RT-PCR amplification or use of new technologies such as NSG have been suggested [14,23,34]. However, high concordance for clinical purposes among the ESTA assay, bulk V3 sequencing, and ultra-deep sequencing has been demonstrated in several studies [5–13,23,35,36], in accordance to our results, which showed similarity in predictions of 80.2%-81.6% with ESTA and 70.6%-77.6% with NGS. In addition, in our study, the use of triple amplification and NGS also showed higher X4 detection rates than single amplification too, but this higher sensitivity did not have clinical significance. On the other hand, the effect of triple amplification on the number of candidates for MVC was striking. First, 14% of the samples could not be amplified in triplicate, similarly to other works [37,38]. Further on, triple amplification concomitant to the use the FPR cut-off of 10% would have excluded in average 25% patients for MVC treatment when compared to single amplification and FPR cut-off = 5%, and in spite of their subsequent response to it. The absence of safety improvement against key increases in costs, turnover time, and number of patients excluded for MVC prescription has resulted in the absence of triple amplification recommendation within the 2014 update of the Austrian-German Treatment Guidelines [15].

Clinical studies have reported an increased R5→X4 tropism switch rate under MVC treatment and detection of X4 viruses as the major reason for treatment failure [1,3,12,39]. However, tropism switches, in both directions, have been detected in patients under both non-MVC- as well as MVC-containing therapies [1,3,40–42], as they are likely to be influenced not only by MVC treatment but also by several different viral or host factors such as HLA haplotype, and IL-4, IL-7, CCR2 and CCR5 polymorphisms [43–47]. In this work, we analysed the tropism switch rate and the effect of X4 viruses detection in nineteen patients after virological rebound, compared to viral sequences from sixteen patients succeeding their MVC therapies. Changes in the FPR after MVC treatment failure were observed: decreases in FPR values occurred in 8.3%-16.7% of the failing patients, but also FPR increases were detected in 12.54%-25.0% of them. These switch values are comparable to those from other studies and also to the spontaneous switch rate between screening and therapy start [1,3,7,12,48,49]. In our study, no statistical correlation between detection of X4 viruses under treatment and therapy failure was detected. Indeed, X4 sequences were found in similar proportion in patients failing their therapies and in those succeeding theirs. Similarly, in the MOTIVATE and MERIT studies as well as one work by Reuter and colleagues [7], 43%, 55% and 42%, respectively, of the failing patients still carried R5 viruses. Therefore, we also investigated the role of other factors such as GSS and MVC-resistance mutations for treatment failure. MVC-resistance mutations either at baseline or after virological rebound could not be correlated with therapy failure. Regarding GSS, development of resistance to the concomitant OBT has been described in previous analysis in 17% (MERIT) and 27% [7] of the failing patients. Here, two (10.5%) of our patients probably failed due to absence of active drugs in the OBT. Nevertheless, no correlation between the GSS at baseline or after virological rebound and therapy failure was found, as also reported a previous study [6], but contrary to others [12,48]. This lack of correlation between GSS and outcome in our patients is probably a consequence of another key factor for virological suppression, the adherence to treatment. Therapy adherence is an important problem, in the daily HIV clinical routine [50], especially for heavily- and long-treated patients, as ours were. Indeed, six of our failing patients displayed a higher GSS at therapy failure compared to baseline, suggesting a very poor adherence [51]; indeed for two of these patients poor adherence was confirmed by the treating physician. Viruses from other two failing patients displayed a GSS = 2 (completely susceptible to OBT) and FPR>20, conditions that would lead to virological suppression if the drugs were taken regularly. For four further patients, poor adherence was also confirmed by the treating physician. Taking all these data together, we propose that in our cohort one major reason, if probably not the only, for MVC therapy failure in patients carrying baseline R5 viruses was poor adherence. More works and patient cohorts are needed to deeper analyse of the causes for MVC failure and correlate them with adherence and baseline parameters.

In conclusion, our work analyses the usefulness of population-based sequencing followed by use of the geno2pheno_[coreceptor]_ tool for tropism prediction for patients currently attending HIV day units. Our data suggest that genotypic testing with single amplification from RNA or DNA FPR and use of cut-off = 5.0% would assure a safe administration of MVC without excluding many patients who could benefit from this potent antiretroviral drug that also seems to reduce the immune activation. Promising results have also been obtained for T-CUP that should be confirmed in future works. In addition, we also showed that treatment adherence may be a critical factor for therapy success in patients on MVC-containing therapies with R5 tropism at baseline.
